# PARTNER: Nurse Participation and Professional Experience During Family Conferences in Intensive Care: A Qualitative Study

**DOI:** 10.1111/nicc.70724

**Published:** 2026-10-08

**Authors:** Anaëlle Caillet, Laurent Poiroux, Mathilde Marchal, Anne Termoz, Bernard Allaouchiche, Julie Haesebaert, Auguste Dargent

**Affiliations:** ^1^ Department of Anesthesiology and Intensive Care University Hospital Lyon Sud, Hospices Civils de Lyon Oullins‐Pierre‐Bénite France; ^2^ University of Angers‐University of Rennes, Inserm, EHESP, Irset (Institut de Recherche en Santé, Environnement et Travail), UMR‐S 1085, SFR ICAT Angers France; ^3^ Public Health Department, Clinical Research and Epidemiology Service Hospices Civils de Lyon Lyon France; ^4^ Research on Healthcare Performance (RESHAPE), INSERM U1290, Université Claude Bernard Lyon 1 – Domaine de Rockefeller Lyon France; ^5^ APCSe VetAgro Sup UPSP 2016.A101 Marcy l'Etoile France

**Keywords:** communication, family conference, ICU, nurses, nurses' participation

## Abstract

**Background:**

Effective communication during family conferences (FC) in the intensive care unit (ICU) is essential for supporting families and fostering trust. Although nurses are continuously present at the bedside, their active participation in FC remains limited, structurally constrained and underexplored from the perspective of healthcare professionals.

**Aim:**

This study aimed to explore the factors shaping ICU nurses' participation in family conferences.

**Study Design:**

This French single‐centre qualitative interpretive study used semi‐structured interviews with nurses and physicians. Transcripts were analysed using a thematic approach combining inductive and deductive coding, managed with NVivo software and reviewed by a multidisciplinary team.

**Findings:**

Nineteen HCP participated (14 nurses and 5 physicians). Three axes shaped HCP experience of FC: organizational conditions (setting, time and exclusion from ethical deliberation), interpersonal dynamics with families (comprehension, trust and relational skills) and interprofessional dynamics (nurses' specific role, barriers, facilitators and benefits of their involvement). Nurses did not express dissatisfaction but identified structural constraints limiting their participation, alongside a clear sense of their own legitimacy. Facilitators included clinical experience, physician trust and structured FC. Barriers included limited speaking opportunities, fear of contradicting physicians and exclusion from ethics meetings. When nurses participated actively, HCP perceived consistent benefits: team coherence, richer communication and strengthened family trust.

**Conclusions:**

ICU nurses' participation in family conferences is primarily shaped by organisational and interprofessional conditions rather than by their willingness or competence. Structural barriers limit their active involvement despite consistent recognition of their contribution by both professional groups.

**Relevance to Clinical Practice:**

These findings provide an empirical foundation for developing structured co‐facilitation models between physicians and nurses during family conferences, and highlight key organisational conditions to address in daily ICU practice.

## Impact Statements

- What is known about the topic
  ○ Effective communication between ICU healthcare professionals and families during family conferences is essential for fostering families' trust and supporting their psychological well‐being
  ○ Nurses' role is frequently restricted to emotional support or logistics, with minimal involvement in FC.
  ○ Families value the nurse's presence during and after FC at the bedside as a necessary complement to medical information to ensure clarity and support.
- What this paper adds
  ○ ICU physicians and nurses perceived their roles during family conferences as complementary, with nurses contributing continuity of care, reformulation of medical information and relational support for families.
  ○ Self‐censorship and fear of undermining medical authority are primary psychological barriers to active nursing participation.
  ○ Nursing participation in FC depends more on clinical seniority and interprofessional trust than on formal institutional protocols.

## 1 Introduction

Intensive care unit (ICU) admission of a relative is a major stressor for families, with 30%–70% of family members developing clinically significant anxiety or depression, sometimes evolving into Post‐Intensive Care Syndrome‐Family [1, 2, 3]. Communication quality during family conferences (FC) has been identified as one of the key modifiable determinants of these outcomes [1, 2]. FC are distinct from the informal, continuous communication that nurses maintain with families at the bedside: FC are structured, planned encounters involving the medical team and family members, focused on diagnosis, treatment strategy and prognosis, and conducted in a dedicated setting [4, 5]. Structured FC are recommended by international guidelines as a standard component of ICU care [4, 5], with specific recommendations for end‐of‐life and palliative care contexts [6]. In practice, however, FC are frequently conducted without a standardized protocol, in inappropriate settings and with insufficient time allocated [5, 7].

Most evidence on FC quality concerns family‐reported outcomes and recommendations on FC content and structure [4, 5], while healthcare professionals (HCP) experience of these encounters remains under‐documented. Nurses are the HCP most continuously present at the bedside and are regarded by families as trusted communicators [8], yet their active participation in FC remains limited despite their frequent physical presence [9]. Recent qualitative work shows the cost of this marginal position: nurses report feeling powerless and excluded from decision‐making, alongside communication difficulties with families and moral distress, particularly in end‐of‐life contexts [10]. A scoping review similarly found that nurses bear a disproportionate burden during treatment withdrawal discussions, often without being formally included in the decision‐making process [11]. Nurses are also frequently excluded from ethics meetings or left uninformed when FC take place, which fragments post‐conference family support [12]. A recent scoping review identified care overload, lack of specific communication training and use of technical language as the most frequent barriers to effective nurse‐family communication in the ICU [13]. Conversely, when nurses participate proactively in FC, family anxiety and depression decrease significantly [9] and a nurse‐led family support intervention—while distinct from FC participation—has been shown to improve family emotional outcomes in a randomized trial [14]. Nurses' specific contributions across the preparation, facilitation and follow‐up phases of FC have also been described [15].

This evidence points to a gap that has not yet been bridged: nurses are consistently described as essential to family communication and as bearing a disproportionate emotional burden, yet their participation in FC has no formal, structured role. Three areas require further investigation. First, the barriers and facilitators to nurse participation, as perceived jointly by nurses and physicians, have not been systematically explored; the existing literature documents consequences more than mechanisms. Second, the conditions required for a co‐facilitation model—in which physician and nurse assume coordinated, complementary roles—remain undefined, despite evidence that structured nurse involvement improves outcomes [9, 14]. Third, ICU nurse experience of FC, as distinct from family satisfaction, has received almost no direct empirical attention [8, 13], limiting our ability to design FC models that are sustainable in daily practice rather than dependent on individual physician willingness.

This study aimed to explore the factors shaping ICU nurses' participation in family conferences.

## 2 Design and Methods

### 2.1 Design

This study was a French exploratory qualitative investigation conducted at a single centre. It employed a qualitative interpretive design to examine nurse participation during FC in ICU. This approach assumes that participants' perceptions are shaped by their professional backgrounds and experiences as well as by their interaction with the interviewer. Semi‐structured interviews were conducted to capture the perspectives of HCP, focusing on their experiences, perceived barriers, facilitators and benefits. The data were analysed following the framework described by Miles and Huberman, which involves three concurrent flows of activity: data reduction, data display and conclusion drawing and verification [16]. This article follows the Standards for Reporting Qualitative Research Guidelines [17].

### 2.2 Setting and Sample

The study was conducted in a 30‐bed mixed ICU within a university‐affiliated hospital. FC were typically held in person, often in a designated room and were organized according to clinical urgency and family needs or requests, rather than on a fixed schedule. They were typically attended by the referring physician and the nurse on duty, alongside close family members, with no standardized protocol defining the roles of each participant or the structure of the encounter. Family visits were allowed 24/7; family members had access to a waiting room and, in certain cases, could stay in the patient's bedroom overnight. The patient‐to‐nurse ratio was 2.5:1.

Inclusion criteria were physicians and nurses working in the ICU; HCP with less than 1 year of ICU experience were excluded. We used a convenience sampling strategy, inviting all eligible ICU staff to participate voluntarily via email and a visual awareness campaign displayed in the ICU. To ensure a diversity of perspectives, we aimed for approximately one quarter of the sample to be physicians. Recruitment and interviews were conducted over 3 months in 2025 until data saturation was achieved, defined as the point at which no new codes or themes emerged from successive interviews.

The inclusion of both nurses and physicians provided data source triangulation, elucidating how their complementary viewpoints inform the nursing role in FC and future interprofessional collaboration. Additionally, coding was performed by the principal investigator and reviewed by a social psychologist, ensuring investigator triangulation. Should practices evolve as a consequence of these findings, the endorsement of medical teams would be crucial for implementation.

### 2.3 Researcher Characteristics

The research team comprised the principal investigator (an ICU nurse), a second nurse, a social psychologist, a methodologist, a public health physician and two physicians. All interviews were conducted by an ICU nurse from the same unit, who was also a member of the research team. The main investigator, a female ICU nurse with 8 years of experience and training in qualitative research, conducted the interviews. Her role as both caregiver and researcher was shared with the participants before the interviews. To address this, reflexivity was used throughout the study.

### 2.4 Data Collection Tools and Methods

The HCP interested in the study contacted the research team either by mail or physically. After checking eligibility, they provided written consent for participation and audio recording. The professional set up an interview with the research team at a convenient time, during shifts or during nonworking days. Every HCP who wanted to take part (before saturation data) was interviewed. The interviews were conducted in an office above the ICU. The interview guide (Table 1) was created by the research team based on the existing literature on ICU FC. It was tested with two ICU staff members (not in the final sample) to ensure that it was clear and relevant. Field notes were taken during each interview to capture details, impressions and nonverbal cues, which were added to the verbatim transcripts for analysis. The information collected included demographic data, years since graduation, professional experience and extra training. Each interviewee received a pseudonym before the session. The interviews were recorded and transcribed by two research team members.

**TABLE 1:** Topics covered in the interview guides.

Topics	Sub‐topics
1. Sociodemographic and professional characteristics	Age, sex
	Year of diploma or end of residency
	Medical specialty
	Seniority in the department
	Status in the department
	Additional training
2. Organizational Procedures for FC	Location of FC
	Professionals usually present
	Average duration of FC
	Introduction round (presentation of HCP/families)
3. FC Content and structure	Existence of a structuring guiding thread
	Variability of practices among HCP
	Type of information provided (diagnosis, prognosis, treatments)
4. Nurse participation during FC	Frequency and conditions of speaking
	Topics addressed by nurses
	Factors influencing participation
	Doctors' perceptions of the nurse's role in FC
	Doctors' expectations regarding nurses' participation
5. HCP perceptions of family communication and understanding during FC	Perception of families' understanding
	Experiences of misunderstandings after FC
	Perceptions of barriers to understanding
	Differences between family communication during FC and at the bedside
6. HCP experience of FC	Evaluation of experience according to FC type (telephone, in‐room, formal, end‐of‐life…)
	Determinants of positive and negative experience
	Perceptions of optimal FC organization according to professionals

### 2.5 Data Analysis

All transcripts were anonymized and transcribed verbatim. Data analysis followed the three‐stage framework of Miles and Huberman [16]. During data reduction, transcripts were coded using NVIVO software (QSR International, USA): deductive codes were derived from the interview guide, while inductive coding allowed new insights to emerge. During data display, codes were compared iteratively and grouped into categories, then synthesized into overarching themes (Table 2). During conclusion drawing and verification, coding was performed by the principal investigator and reviewed by a social psychologist, ensuring consistency across the analytical process. Data saturation was achieved after 17 interviews and confirmed by two additional interviews. Participant characteristics were reported using absolute numbers (percentages) or as medians with interquartile ranges [IQR]. Verbatim quotes were originally collected in French and translated into English by the research team; translation accuracy was verified by a bilingual researcher external to the study.

**TABLE 2:** Thematic structure of the PARTNER study: Themes, categories and codes derived from inductive‐deductive analysis of semi‐structured interviews (*n* = 19).

Theme/Category	Codes
Theme 1 — Analysis of FC practices	
A. The second interview	Presence/absence of a distinct paramedical interview after the medical interview
B. Family in the ICU	B1. Needs · B2. Comprehension · B3. Emotional state · B4. Fears
C. FC content & format	C1. Structure · C2. Legislation · C3. Types of FC
D. Nurse participation	D1. Frequency & modalities · D2. Professional perspectives
E. Healthcare assistant role	Presence/absence during FC
F. Physician status	Hierarchical and symbolic positioning during FC
G. Patient's place	Patient presence and involvement during FC
Theme 2 — Nurse's role and participation during FC	
H. Nurse's role	H1. Delivering well‐being · H2. Knowledge · H3. Trust · H4. Legitimacy · H5. Proximity · H6. Daily life · H7. Reformulation · H8. Reassurance · H9. Witnessing information · H10. Life in the ICU
I. Facilitators of speaking	I1. Physician trust · I2. Patient record knowledge · I3. Experience · I4. Explicit invitation · I5. Caregiver personality · I6. Feeling legitimate · I7. FC structure
J. Barriers to speaking	J1. Physician refusal · J2. Limited record knowledge · J3. Lack of anticipation · J4. Judgement · J5. Repetition risk · J6. Trust relationship · J7. Fear of discrediting physician · J8. Limited experience · J9. Conflicts · J10. Caregiver emotions · J11. Gender · J12. Nonverbal cues · J13. Responsibility · J14. Admission interview · J15. Legitimacy · J16. No family questions · J17. Time pressure
K. Benefits of nurse speaking	K1. Team cohesion · K2. Paramedical credibility · K3. Physician support · K4. Humanity · K5. Proximity · K6. Patient record understanding · K7. Family trust · K8. Complementarity
Theme 3 — Determinants of negative HCP experience during FC	
L. FC preparation & format	L1. Frequency · L2. Ethics meetings · L3. Nurse not invited · L4. Workload · L5. Structure · L6. Location · L7. Time pressure
M. Caregiver‐related factors	M1. Minimizing severity · M2. Judging family reactions · M3. Nurse not speaking · M4. Limited record knowledge · M5. Opaque decisions
O. Family & external factors	O1. Emotional state · O2. Absence of questions · O3. Task interruption · O4. Misunderstanding · O5. Conflicts
Theme 4 — Determinants of positive HCP experience during FC	
P. FC content, format & preparation	P1. Location · P2. Participants · P3. Duration · P4. Anticipation · P5. Content · P6. Face‐to‐face contact
Q. Nurse‐related factors	Q1. Experience · Q2. Patient record knowledge · Q3. Reformulation
R. Family‐related factors	R1. Emotional state · R2. Comprehension · R3. Questions · R4. Expression of gratitude
S. Relational skills	S1. Trust · S2. Dialogue · S3. Benevolence · S4. Active listening · S5. Consensus · S6. Teamwork · S7. Communication

### 2.6 Rigour

Rigour was established according to Lincoln and Guba's criteria for trustworthiness [18]. Credibility was supported by investigator triangulation (coding by the principal investigator and reviewed by a social psychologist). Data source triangulation (dual nurse and physician perspectives), prolonged engagement with the data and reflexivity were maintained throughout the research process. Transferability was facilitated by a detailed description of the study context and participant characteristics, enabling readers to assess applicability to other settings. Dependability was ensured through the systematic use of the Miles and Huberman analytical framework, the use of NVIVO software for data management and the pre‐testing of the interview guide. Confirmability was supported by the use of verbatim quotes to ground interpretations in participants' own words, and by peer review of the analytical process by a researcher external to the ICU setting.

### 2.7 Ethics Statement

This study received CNIL approval from the Hospices Civils de Lyon under number 24–125 (February 20, 2025), as well as approval from the Hospices Civils de Lyon ethics committee and is registered under number IRB00014232.

## 3 Findings

Nineteen HCP participated (14 nurses, 5 physicians), with a median age of 30 years [25.5–35] and median ICU seniority of 3 years [2.0–4.5]. Recruitment took place between March and June 2025. Six nurses reported never speaking during FC; two physicians never allowed nurses to do so (Tables 3 and 4):

There were physicians who did not want us to speak at all. Who did not want us to intervene. It never really stopped me from doing it (smiling). Some of them, it's like ‘I am a physician, you are a paramedic’. The physician is the one who knows. (Nurse no. 2)

**TABLE 3:** Characteristics of participants.

Participant characteristics	Nurses (*n* = 14)	Physicians (*n* = 5)
Age, median [IQR]	27.5 [25–35]	35 [33.5–35]
Year since graduation^a^, median [IQR]	5.5 [3–8]	5 [4–5.5]
Years in ICU, median [IQR]	4.0 [2.5–5]	3 [2.5–4.5]
Women, *n* (%)	10 (71%)	1 (20%)
ICU certification, *n* (%)	9 (64%)	N/A

^a^ For nurses: years since nursing diploma. For physicians: years since end of residency.

**TABLE 4:** Individual characteristics of participants.

Participants	Job	Age	Sex	Years since graduation	Years in ICU
1	Nurse	37	F	16	4
2	Nurse	48	M	17	16
3	Nurse	30	F	6	6
4	Physician	34	F	5	3
5	Physician	35	M	6	2
6	Physician	35	M	5	5
7	Nurse	30	F	8	5
8	Nurse	24	M	3	5
9	Physician	35	M	3	3
10	Nurse	25	F	3	3
11	Nurse	22	M	1.5	1
12	Nurse	28	F	6	2
13	Physician	33	M	5	4
14	Nurse	35	F	11	2.5
15	Nurse	27	F	5	5
16	Nurse	26	M	4	4
17	Nurse	25	F	3	1
18	Nurse	36	M	7	4
19	Nurse	23	F	4	2.5

Three themes emerged from the analysis: (a) organizational conditions shaping the encounter: setting, time and exclusion from ethical deliberation; (b) interpersonal dynamics with families: comprehension, emotional climate and relational skills; and (c) interprofessional dynamics including nurses' specific role, barriers to their participation and the benefits of their involvement. Together, these themes point towards a co‐facilitation model between physician and nurse.

- Organizational conditions influencing nurse participation in family conferences (Figure 1).

**FIGURE 1:**
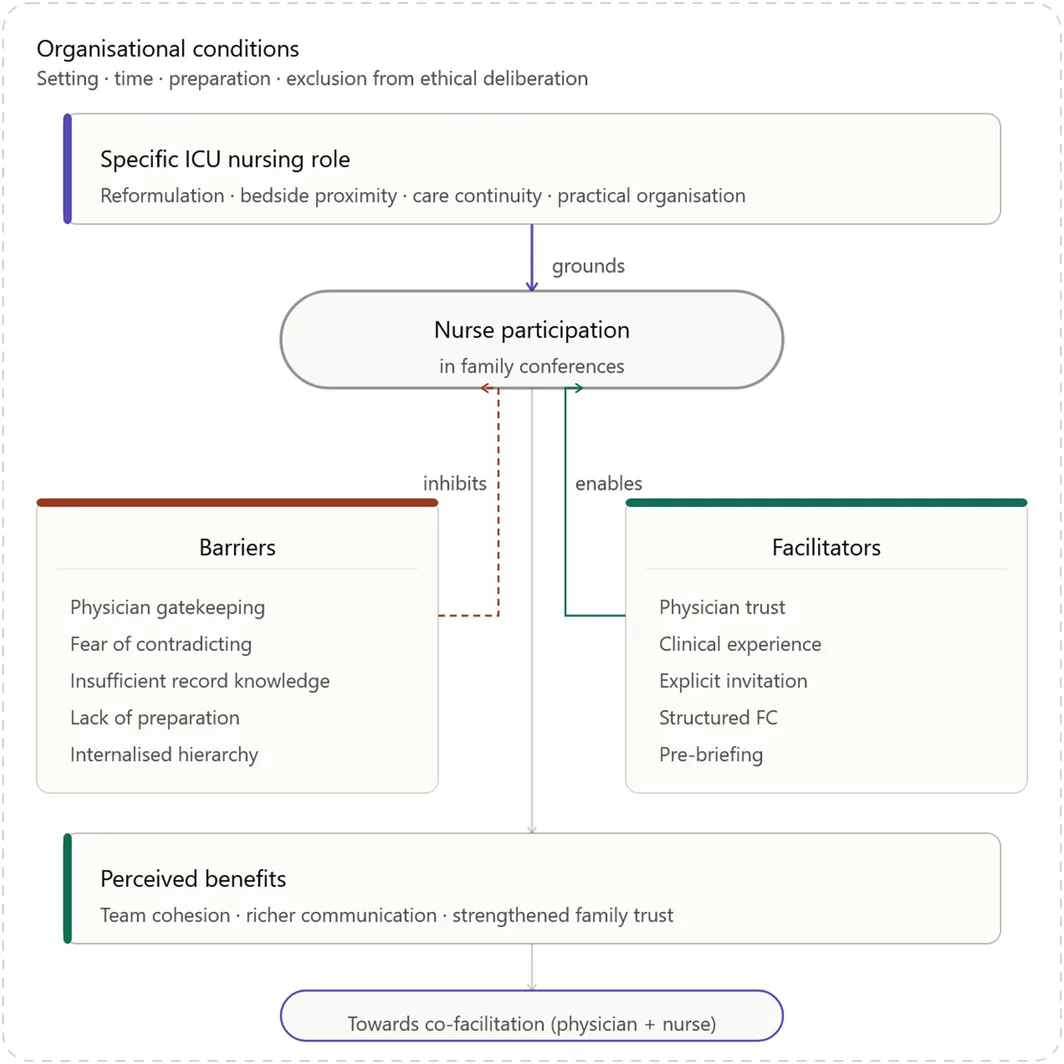
Organizational and interprofessional conditions shaping nurse participation in ICU family conferences and perceived benefits of their involvement.

Experience with FC was primarily shaped by structural conditions preceding and framing the encounter, independent of individual communication skills.

### 3.1 Setting, Composition and Structure

The physical setting was the most consistently cited factor. A dedicated, quiet room was identified as a prerequisite for quality exchange, while the patient's bedside was widely considered inappropriate—HCP perceived families as distracted by their surroundings and unable to concentrate on the information given. FC conducted by phone were uniformly viewed as inadequate. The composition of the FC also mattered: participants favoured limiting attendance to close relatives, the most involved physician and the nursing staff on duty. Within the encounter itself, a clear and structured format was perceived by HCP as supporting comprehension and reducing the risk of confusion for both families and themselves:

It's important to be clear and organized. When I feel a bit lost during the interview, I cannot even imagine the family. (Nurse no. 12)

### 3.2 Time, Workload and Preparation

Temporal organization was identified as a critical yet structurally under‐resourced condition. High workload directly eroded the quality of HCP presence during FC, shifting the encounter from a relational act to a task to complete. Anticipation—informing families in advance and allowing preparation time—was perceived as a counterweight to this pressure, enabling more considered and responsive communication:

I am less patient because I have work to do, the mental load of the day. If it is a time when there is a bit of break, you can take more time. (Nurse no. 18)

### 3.3 Exclusion From Ethical Deliberation

A recurring and specifically nurse‐reported source of negative experience was exclusion from ethics meetings and, in some cases, from FC itself. This exclusion had direct consequences for care continuity: nurses encountered families with unresolved questions about decisions they had not been part of, undermining their capacity to provide coherent support. More broadly, absence from ethical deliberation left nurses without clarity on therapeutic strategies, positioning them at the margins of the care pathway they were otherwise central to:

It's happened more than once. They went to a meeting, and I did not even know. And then you are faced with questions from the family, and you cannot even answer them. (Nurse no. 17)

Nurses are not necessarily part of ethical discussions. In the end, nurses don't know what will be the patient future. (Nurse no. 10)

b Interpersonal dynamics influencing nurse participation in family conferences (Figure 1).

Beyond structural conditions, the perceived quality of the experience was co‐produced through the relational dynamics of the encounter.

### 3.4 Comprehension and Dialogue

Family comprehension was identified as the primary marker of a successful FC: HCP experience depended on the sense that information had been understood, not merely delivered. Equally, space for families to ask questions and express themselves was valued as a condition for genuine dialogue rather than one‐directional transmission. Conversely, conflictual situations—whether between family members or with the team—and environmental disruptions consistently diminished this relational quality:

It's true that if they did not understand gravity, I would really feel like I did not achieve the interview aim. (Physician no. 4)

### 3.5 Trust and Relational Skills

The relational dimension of the encounter—the capacity to build trust, demonstrate empathy and sustain attentive presence—was the factor most consistently associated with a sense of a well‐conducted FC. Trust was experienced as the defining marker of success, distinct from informational content. Reaching a shared consensus with the family on the therapeutic strategy added a further dimension of professional fulfilment, confirming that communication had been not only clear but effective:

What is most striking at the end of an interview is when the family says they trust us. This is perhaps the element that will most determine my feeling of a well‐conducted interview. (Physician no. 6)

Nurses were particularly attentive to physician communication style, which they perceived as immediately setting the relational tone of the entire encounter—a finding that points to the interprofessional dimension of family communication.

c Professional roles and interprofessional factors shaping nurse participation in family conferences.

The data revealed a central tension: nurses hold a recognized and specific contribution to FC—grounded in bedside proximity, reformulation skills and continuity of care—yet their participation remains structurally constrained by hierarchical dynamics and insufficient preparation. Nurses did not express strong dissatisfaction with their current role, but rather identified the conditions that limit their participation and articulated a clear sense of their own legitimacy, pointing towards ways to enhance their contribution.

### 3.6 The Distinctive Role of Nurses

Nurses' sustained bedside presence and intimate knowledge of patients constituted the foundation of their specific contribution to FC. Their capacity to reformulate medical information in accessible language was identified by most participants as one of the most valuable functions nurses can serve during or after FC—perceived as enabling families to consolidate understanding in a way that complements, rather than duplicates, physician communication. Beyond reformulation, nurses manage the logistical and relational dimensions of the ICU environment that families encounter daily, functions that HCP described as experienced by families as forms of continuity and welcome:

Clearly, we spend the whole day by the patient's bed. The physician will spend a good part of his time there, but he will not have that closeness, that intimacy that we will have with the patient. (Nurse no. 10)

### 3.7 Barriers: Hierarchy and Internalized Inhibition

Despite this recognized contribution, nurse participation was systematically limited. The primary barrier was structural: physicians frequently failed to create space for nurses to speak and some actively discouraged it. Beyond explicit exclusion, nurses described internalized inhibitions rooted in professional hierarchy—fear of contradicting the physician, of undermining medical authority or of being judged for exceeding the perceived boundaries of nursing competence. These fears operated as self‐imposed silences even in the absence of direct prohibition and constituted a distinct barrier to interprofessional communication:

I'm afraid of discrediting the physician, even though in the end it's complementary. […] I think you're a bit afraid of scratching the physician's ego. (Nurse no. 3)

### 3.8 Facilitators: Trust, Experience and Structured Invitation

Nurse participation was enabled when physicians explicitly created space for nursing input—most commonly by inviting a contribution at the close of the FC—and when mutual trust between physician and nurse had been established through prior collaboration. Clinical experience also played a central enabling role: nurses with greater ICU seniority reported greater confidence in the legitimacy of their participation. A well‐structured FC with prior role coordination made intervention considerably easier:

Some physicians ask me: “Do you have anything to add?” Not when they're talking, but afterwards. And so, at that moment, there are indeed things that can be said. (Nurse no. 17)

### 3.9 Towards Co‐Facilitation: Benefits and Perspectives

When nurses participated in FC, HCP perceived several consistent benefits. First, joint participation was described as conveying team coherence to families—communicating that decisions were collective and that the family was engaging with a unified team. Second, complementary contributions from both professionals produced richer communication than either could achieve alone, provided roles had been anticipated and coordinated in advance. Third, nurse participation was perceived as strengthening family trust. The nurse's presence throughout the day and at the FC was described as reinforcing a sense of sustained, personalized attention for families:

It adds trust in the relationship with the family, the fact that we're there in the room all day, that we're here with the physician for the interview. (Nurse no. 3)

Together, these benefits point towards a co‐facilitation model in which physician and nurse assume deliberate, coordinated roles within FC. This would require, as participants emphasized, prior preparation and institutional legitimization of nursing participation—not as a residual addition, but as a structured component of interprofessional communication in the ICU.

## 4 Discussion

The central finding of this French study is that, although ICU nurses are widely recognized as essential contributors to FC, they remain structurally marginalized in their participation. This finding is consistent with previous research [8, 13], while providing new insights into the mechanisms that sustain this gap. The structural nature of this gap is confirmed by the primacy of organizational conditions in shaping HCP experience: setting, time and exclusion from ethical deliberation were the most consistently cited determinants, regardless of professional role, echoing evidence that FC quality is first an organizational problem before being a relational one [4, 5]. At the interpersonal level, family comprehension and trust emerged as the defining markers of a well‐conducted FC for HCP, consistent with family‐reported evidence [1, 9] and suggesting that the standards professionals set for themselves align with what families value. One finding adds nuance to existing recommendations: while the literature consistently advocates for a dedicated, quiet room [4, 5, 19, 20], nurses in this study reported greater communicative ease at the bedside, suggesting that their relational practice with families develops in a different spatial register than formal FC and that future protocols should account for this distinction.

A central finding is what Debue [21] describes as the ‘structural invisibility’ of nurses within the interprofessional dyad. Nurses are physically present during FC but often functionally absent: excluded from ethics meetings, sometimes uninformed when FC take place and rarely invited to speak. Importantly, this silence does not reflect a lack of willingness or competence. Nurses in this study clearly articulated what they would contribute and why they felt legitimate to do so. Rather, it reflects a structural constraint: without institutional space and physician invitation, even motivated nurses default to silence to avoid interprofessional conflict. This exclusion generates an informational deficit—nurses encounter families with questions about decisions they were not part of, undermining their capacity to act as a semantic relay between the medical team and the family. This fragmentation of care continuity is particularly consequential in end‐of‐life FC, where nurses reported the lowest levels of professional experience and where the absence of multidisciplinary deliberation generates misunderstandings that HCP perceived as leading to diminished communication quality for families [7]. These findings extend those of Kawashima et al. [15], who described nurses' contributions across three FC phases—preparation, facilitation and follow‐up—by demonstrating that these contributions are contingent on prior access to ethical deliberation. When nurses are excluded from ethics meetings, their ability to prepare for and follow‐up on FC is structurally compromised. The tension between care time and communication time identified in this study further suggests that nursing agency in FC remains anchored in clinical proximity rather than in the formal spaces of medical decision‐making—a dynamic rooted in professional hierarchy rather than competence.

Barriers to nurse participation operate at two distinct levels. At the structural level, physicians frequently failed to create space for nursing input, and some actively discouraged it—a pattern consistent with recent evidence on interprofessional dynamics in ICU teams [13]. At the individual level, nurses described internalized inhibitions: fear of contradicting the physician, of undermining medical authority and of exceeding the perceived boundaries of nursing competence. Crucially, these inhibitions coexisted with a clear sense of legitimacy. Nurses knew what they had to offer and why their contribution mattered, but chose silence as a strategy to preserve interprofessional relationships rather than out of genuine uncertainty about their role. This dynamic is further compounded by workload: in high‐demand ICU contexts, family engagement—including participation in FC—is among the nursing activities most frequently deprioritized when workload becomes excessive, as nurses prioritize physician‐prescribed tasks over relational care [22]. These self‐imposed silences are reproduced even in the absence of explicit prohibition, echoing the service culture that sacralizes medical speech identified by Debue [21]. The literature provides limited insights into these factors, with clinical experience being the only facilitator consistently documented [9, 15, 23]. Our findings extend this observation and identify two additional facilitators: explicit physician invitation at the close of the FC, and mutual interprofessional trust built through prior collaboration. These findings suggest that enabling nurse participation requires deliberate relational and organizational conditions—not only individual development—including systematic pre‐briefing and role coordination prior to FC.

When nurses participated actively in FC, three consistent benefits emerged: team coherence, richer communication through reformulation and strengthened family trust. These results converge with evidence from Garrouste‐Orgeas et al. and the FICUS trial that structured nurse involvement improves family outcomes [9, 14]. Taken together, these benefits constitute the empirical foundation for a co‐facilitation model in which physician and nurse assume deliberate, coordinated roles within FC. Implementing such a model would require institutional legitimization of nursing participation as a standard component of FC alongside systematic pre‐briefing to define roles and formal inclusion of nurses in ethics meetings to ensure informational continuity. These conditions are consistent with the 2024 ESICM guidelines recommending structured FC and interdisciplinary communication training [6], and address the structural roots of the barriers identified in this study. Nurses are not absent from FC because they have nothing to contribute. They are absent because no one has yet made space for them.

### 4.1 Recommendation

This study generates several hypotheses that warrant further investigation. Whether the structural barriers identified here, particularly physician gatekeeping and exclusion from ethical deliberation, causally limit communication quality and family trust remains to be tested in prospective designs. The conditions under which a co‐facilitation model is feasible and effective across different ICU contexts also require empirical evaluation. Future studies should incorporate the direct perspective of families to assess whether the benefits of nurse participation perceived by HCP translate into measurable improvements in family outcomes.

### 4.2 Limitations

This study was assessed according to Lincoln and Guba's criteria [18]. Credibility was supported by investigator and data source triangulation, reflexivity maintained throughout and prolonged engagement with the data. However, interviews conducted by a nurse from the same ICU may have introduced social desirability bias, despite facilitating rapport. Transferability is limited by the single‐centre design and convenience sampling, which may not reflect the diversity of ICU contexts. The sample was relatively young and had limited ICU experience, which may limit transferability to more experienced teams where interprofessional dynamics and communication practices may differ. However, participants were representative of the overall composition of the ICU team in terms of age, seniority and professional distribution, suggesting that findings reflect the actual practice context rather than a selected subgroup. The detailed description of the setting and participant characteristics nonetheless enables readers to assess applicability to other settings. Dependability was ensured through the systematic use of an established analytical framework and peer review of the coding process. Confirmability was supported by the use of verbatim quotes grounding interpretations in participants' own words. A further limitation is that family experience is captured solely through HCP accounts.

### 4.3 Implication for Practice

FC should be standardized and conducted in quiet, private spaces away from the bedside [4, 5, 7]. A systematic pre‐briefing involving physician, nurse and healthcare assistant should define each participant's role before the encounter. Nurse participation must be institutionally legitimized and not left to individual physician discretion. Communication skills training should be integrated into formal nurse education, not left to accumulate through experience alone. Nurse workloads should be monitored, as high patient‐to‐nurse ratios directly reduce the time available for family communication [22, 24]. The FICUS trial demonstrated that dedicated nurse positions for family support significantly improve family emotional outcomes [14]—a model worth extending.

## 5 Conclusion

This qualitative study suggests that ICU nurses' participation in family conferences is primarily shaped by organizational and interprofessional conditions rather than by their willingness or perceived legitimacy. Nurses' proximity to patients, communication skills, and contribution to continuity of care were consistently recognized by both professional groups, yet structural barriers limited their active involvement. These findings highlight key organizational conditions that may enable meaningful nurse participation and provide empirical insights to inform the development and evaluation of structured physician–nurse co‐facilitation models in ICU family conferences.

## Author Contributions

Study conception and design: all authors. Data collection: A.C. Data analysis and interpretation: A.C., M.M. Drafting of the article: A.C. Critical revision of the article: M.M., A.T., B.A., J.H., A.D., L.P.

## Funding

The authors have nothing to report.

## Conflicts of Interest

The authors declare no conflicts of interest.

## Data Availability

The data that support the findings of this study are available on request from the corresponding author. The data are not publicly available due to privacy or ethical restrictions.
